# Static micromechanical measurements of the flexural modulus and strength of micrometre-diameter single fibres using deflecting microcantilever techniques

**DOI:** 10.1038/s41598-024-53082-4

**Published:** 2024-02-05

**Authors:** Ali Reda, Steve Arscott

**Affiliations:** University of Lille, CNRS, Centrale Lille, University Polytechnique Hauts-de-France, UMR 8520-IEMN, F-59000 Lille, France

**Keywords:** Mechanical engineering, Characterization and analytical techniques, Plant sciences

## Abstract

The mechanical properties of natural and man-made fibres ultimately govern the robustness of products. Examples range from textiles to composite materials for mechanical parts in emerging technological applications. An accurate determination of the mechanical properties of microscopic single fibres is therefore important. Today, macroscopic mechanical techniques, such as tensile testing, are commonly employed to obtain this information. However, a relatively high dispersion of results is often encountered due to a relatively long sample size. As an alternative to tensile methods, we demonstrate here micromechanical techniques to accurately measure the flexural modulus and strength of micrometre-sized diameter fibres without the need of force sensing. To demonstrate our ideas, we use the example of single natural fibres (*Linum Usitatissimum*). The flexural modulus of the single fibres is first accurately measured in the low deflection regime of an inclined bending cantilever in an original setup. The flexural strength of the single fibres is then measured in the high deflection regime of a bending cantilever. Interestingly, the novel measurements have allowed the authors to quantify the flexural strength of two different failure modes in flax fibre, enabling a contribution to plant mechanics.

## Introduction

A precise determination of the mechanical properties of single fibres is important to produce optimised textiles, composite materials, and emerging technologies^1–4^. There are a number of methods employed to characterise single fibres having microscopic dimensions. Today, traditional tensile testing^5,6^ of single fibres is the most common technique to measure the modulus and the strength^7^. However, applying these techniques to such thin single fibres is challenging as it can result in a large dispersion of results^8^, meaning trends in data can be hard to interpret. Therefore, innovative measurement techniques, e.g. using miniaturisation, need to be developed to increase accuracy. Miniaturised tensile testing^9,10^ can be developed using Microelectromechanical systems (MEMS)^11–13^. In these approaches, sample dimensions can be reduced^14^, but an accurate measurement of force^15^ remains challenging.

**Figure 1:**
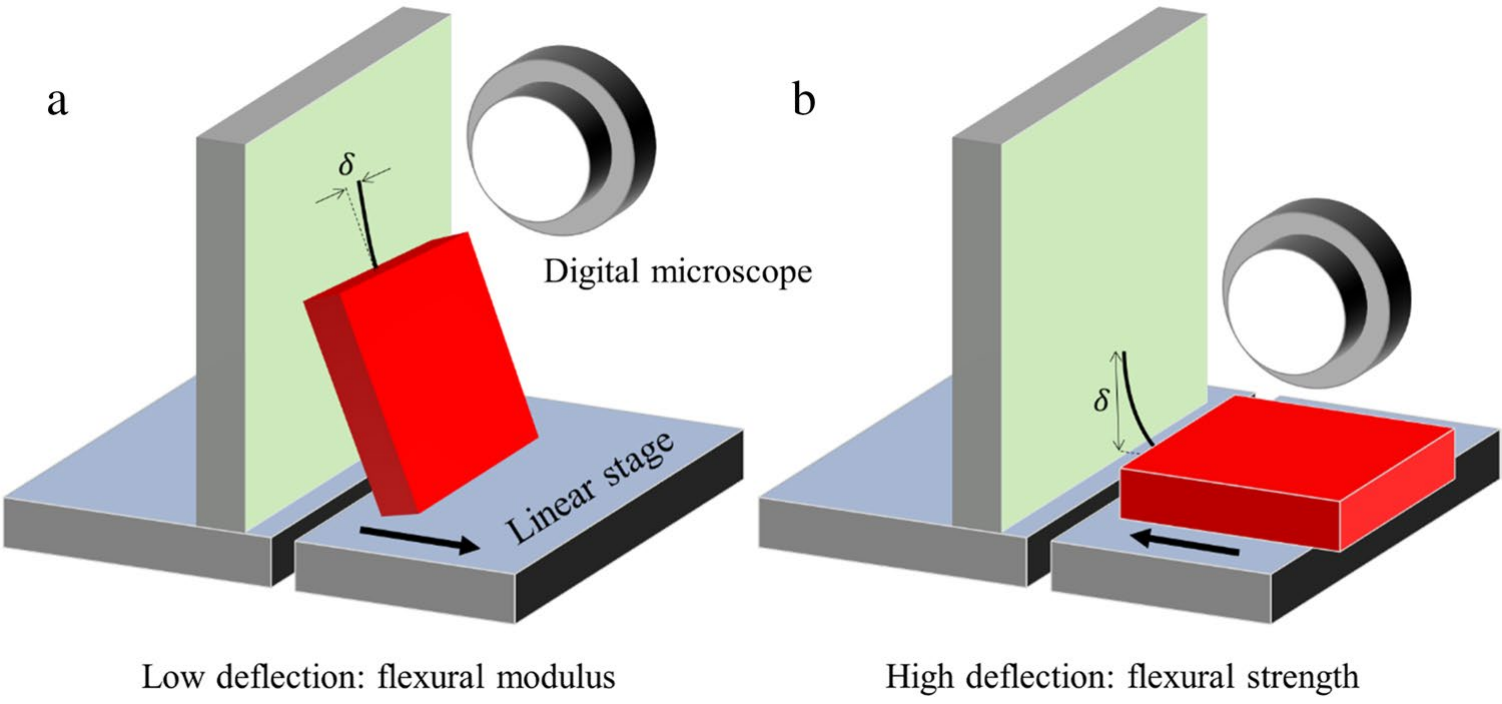
Schematic diagrams showing the two micromechanical measurements developed in the study. (**a**) Low deflection of a fibre-based microcantilever to measure the flexural modulus. (**b**) High deflection of a fibre-based microcantilever to measure the flexural strength. The support chip (red) is moved using a precision linear stage (black arrows).

Here, we demonstrate two cantilever-based micromechanical methods, based on original modelling, to accurately determine the flexural modulus and flexural strength of micrometre-sized diameter single fibres having millimetre lengths. Unlike other micromechanical approaches, no direct measurement of the force is required, i.e. no sensor is needed. The force is determined from a micromechanical model using system parameters (mass, dimensions, and angles) that are easily and precisely measured. Figure 1 shows the two measurement techniques developed in the study.

Figure 1a shows how the weight of the support chip (red) causes the microcantilever to deflect with the tip sliding down the smooth vertical surface (green). The leaning angle of the support chip can be varied using a precision linear stage to vary the deflecting force. The flexural modulus can be determined by plotting the deflection as a function of force. Figure 1b shows how the microcantilever can be put into high deflection using the linear stage by causing the tip of the cantilever to slide up the smooth vertical surface (green). The flexural strength can be determined by measuring the curvature of the fibre just prior to failure.

## Measuring the flexural modulus of a single fibre using a bending cantilever in low deflection

### Model

**Figure 2:**
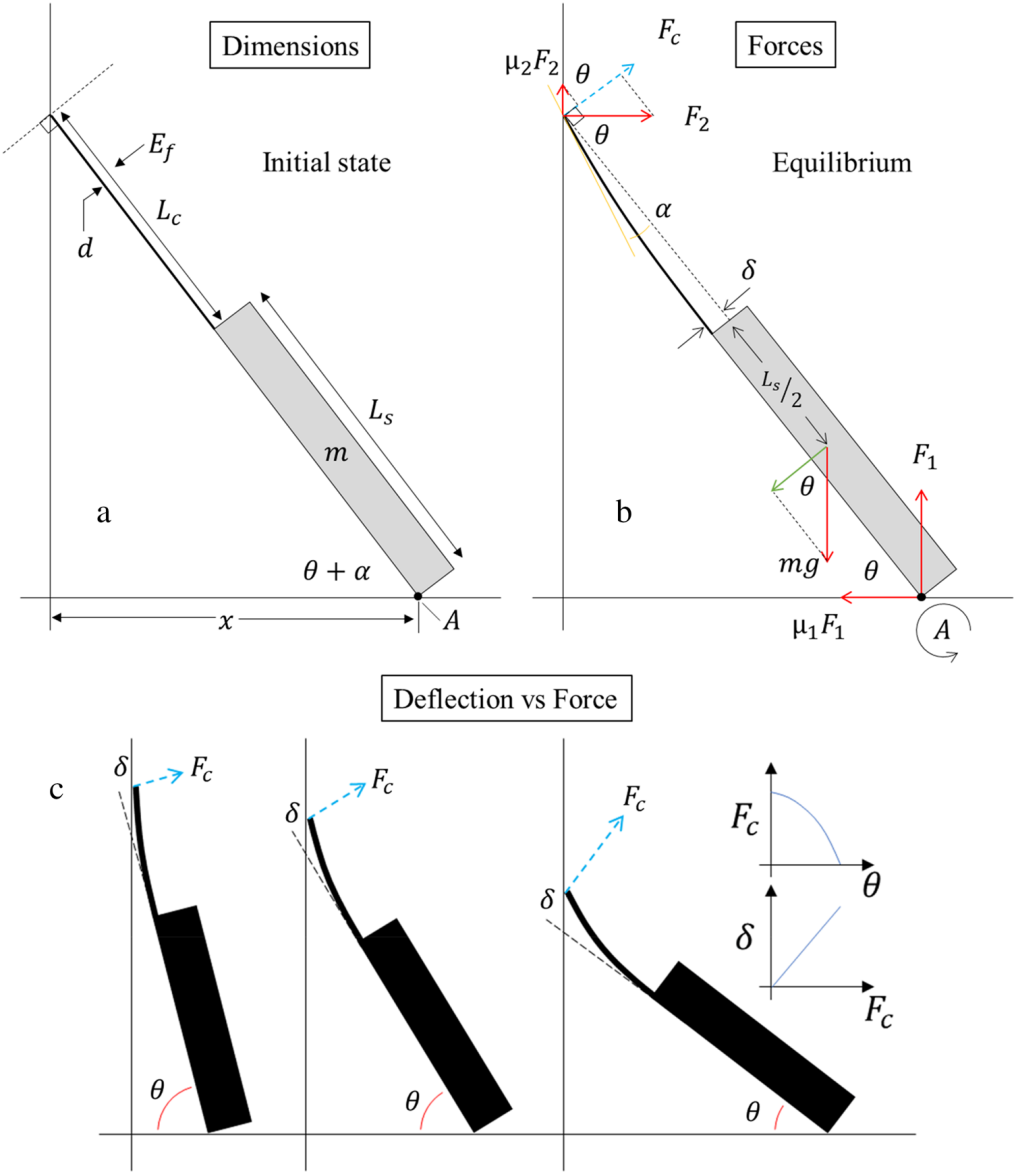
A schematic diagram showing the cantilever/support chip ensemble leaning against a vertical surface. (**a**) Initial state of cantilever/support and relevant dimensions of the system. (**b**) Equilibrium state and the forces acting on the system upon bending of the cantilever in low deflection, and (**c**) The support chip angle governs the bending force (blue arrow) causing the cantilever deflection to vary.

This part of the study involves original modelling of a leaning, deflecting single fibre-based cantilever to measure its flexural modulus. Figure 2 shows a schematic diagram of the cantilever/support chip ensemble leaning against a vertical surface.

We consider a cylindrical cantilever with a uniform cross section attached to a rigid support chip. The cantilever has a diameter $d$ and length ${L}_{c}$. The support chip has length ${L}_{s}$ and mass $m$. The mass of the cantilever is considered negligible compared to the mass of the support chip. The modelling considers friction forces at the cantilever/wall contact point and the support chip/surface contact point. As the cantilever is not rigid, the initial state illustrated in Fig. 2a is not in equilibrium. The cantilever bends and the cantilever/support ensemble will reach a static equilibrium shown in Fig. 2b. In this case, the sum of all forces is zero (Newton’s first law). In equilibrium, the support chip makes an angle with the horizontal surface. The tip of the cantilever will deflect by distance $\delta$. The extent of this bending will depend on all other parameters—including the flexural modulus ${E}_{f}$ of the cantilever’s material.

We can therefore write down all the forces acting on the cantilever/support ensemble–see Fig. 2b. The force due to gravity acting on the support mass will be $m$ g, the normal force on the support chip at point $A$ will be ${F}_{1}$, the frictional force acting at point $A$ parallel to the horizontal surface is given by ${\mu }_{1}{F}_{1}$, and the normal force of the vertical surface acting on the tip of the cantilever will be ${F}_{2}$, together with a parallel frictional force ${\mu }_{2}{F}_{2}$. Where ${\mu }_{1}$ is the static coefficient of friction between the support chip and the horizontal surface; ${\mu }_{2}$ is the static coefficient of friction between the fibre and the vertical surface.

Resolving forces vertically we have:

1 $${F}_{1}+{\mu }_{2}{F}_{2}-mg=0$$

Resolving forces horizontally we have:

2 $${F}_{2}-{\mu }_{1}{F}_{1}=0$$

Taking moments about point $A$ we have:

3 $${F}_{2}{\text{sin}}\left(\theta \right)\left({L}_{s}+{L}_{c}\right)+{\mu }_{2}{F}_{2}{\text{cos}}\theta \left({L}_{s}+{L}_{c}\right)-mg{\text{cos}}\theta \frac{{L}_{s}}{2}=0$$

4 $${F}_{2}=\frac{mg{L}_{s}}{2\left({\text{tan}}\theta +{\mu }_{2}\right)\left({L}_{s}+{L}_{c}\right)}$$

The bending force ${F}_{c}$ (blue dashed arrow in Fig. 2b) perpendicular to the support chip plane is given by:

5 $${F}_{c}={F}_{2}({\mu }_{2}{\text{cos}}\theta +{\text{sin}}\theta )$$

Therefore, ${F}_{c}$ can be written as:

6 $${F}_{c}=\frac{mg{L}_{s}({\mu }_{2}{\text{cos}}\theta +{\text{sin}}\theta )}{2\left({\text{tan}}\theta +{\mu }_{2}\right)\left({L}_{s}+{L}_{c}\right)}$$

which simplifies to:

7 $${F}_{c}=\frac{mg{L}_{s}{\text{cos}}\theta }{2\left({L}_{s}+{L}_{c}\right)}$$

Interestingly, this means that ${F}_{c}$ is independent of wall friction. This means that the measurements are independent of friction.

For a cylindrical cantilever the relationship between the force of a concentrated load at the end of the cantilever ${F}_{c}$ and the tip deflection $\delta$ is given by^16,17^:

8 $${F}_{c}=\frac{3\delta \pi {E}_{f}{d}^{4}}{64{L}_{c}^{3}}$$

Therefore, we can equate Eq. 7 and Eq. 8 to give:

9 $$\frac{3\delta \pi {E}_{f}{d}^{4}}{64{L}_{c}^{3}}=\frac{mg{L}_{s}{\text{cos}}\theta }{2\left({L}_{s}+{L}_{c}\right)}$$

The flexural modulus ${E}_{f}$ of the cylindrical fibre can be written as:

10 $${E}_{f}=\frac{32{L}_{c}^{3}mg{L}_{s}{\text{cos}}\theta }{3\delta \pi {d}^{4}\left({L}_{s}+{L}_{c}\right)}$$

where $\delta$ and $\theta$ can be measured from a high-resolution photograph of the bending cantilever.

There are two constraints for the deflection. The first involves the low-deflection validity of the modelling; the second is a geometric criterion concerning the tangency of the tip of the cantilever with the vertical surface.

The modelling assumes that the sine of the bending angle $\alpha$ can be approximated by $\alpha$, i.e. ${\text{sin}}\alpha \cong \alpha$^18^. This has been demonstrated experimentally for various cantilevers^19–21^. The error remains small if:

11 $$\frac{\delta }{{L}_{c}}\le 0.3$$

In terms of geometry and tip tangency with the vertical surface^20,21^:

12 $$\frac{\delta }{{L}_{c}}\le \frac{1}{3}(\pi -2\theta )$$

Equation 12 is derived in the Supplementary Information.

Note that the problem can also be solved for a rectangular cantilever, we give this solution in the Supplementary Information for other applications, e.g. finding the stiffness of a microcantilever.

We can now see that by varying the perpendicular distance $x$ (Fig. 2a) of the support chip from the vertical surface, the leaning angle $\theta$ will vary and modulate the force ${F}_{c}$ in a controlled manner. Increasing ${F}_{c}$ increases the deflection—this is shown schematically in Fig. 2c.

### Experiments and results

**Figure 3:**
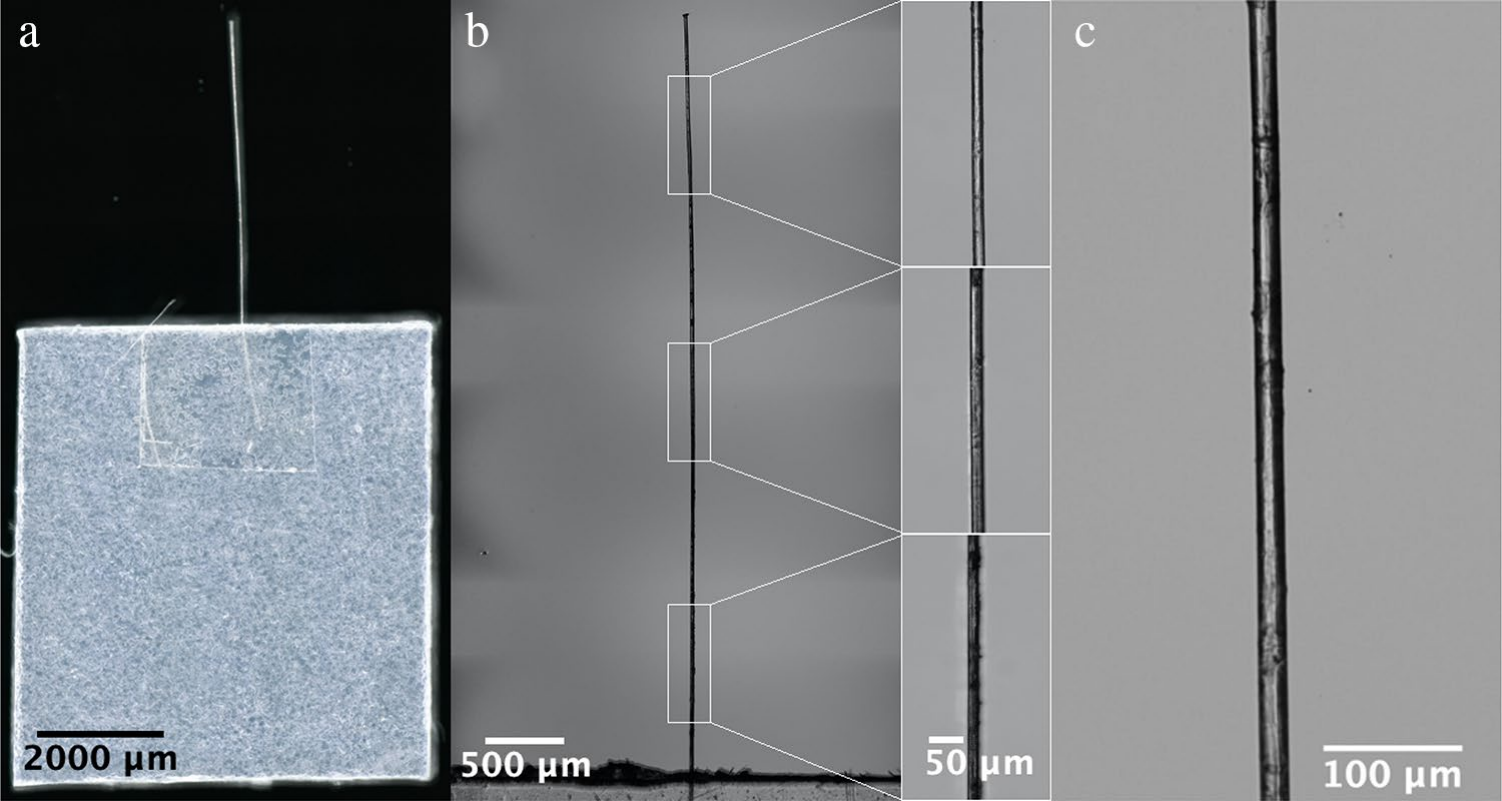
Optical images of the single flax fibre-based cantilever/polypropylene support chip used for the low-deflection measurements. (**a**) Stitched plan view, (**b**) top view of cantilever and (**c**) zoom of the side view of the cantilever.

Single flax fibres, having lengths of ~ 1 cm, were carefully extracted from flax stems using a number of in-house methods developed by the authors (see “Methods” section). The experiments were conducted in an ISO 7 class cleanroom (see “Methods” section). Under these conditions, flax fibres have a moisture content of approximately 10%^22^. A dynamic measurment of the same fibres showed that the viscoelasticity is negligible^23^. Figure 3 shows optical microscope images of a cantilever/support chip ensemble fabricated for the study. The chips are composed of single flax fibres attached to square polypropylene support chips. The details of the fabrication are given in the “Methods” section.

Figure 3a shows a top view of an example of a whole cantilever/polypropylene chip fabricated for the study. Correct perpendicular alignment of the cantilever with the support chip during fabrication ensures that the cantilever is vertically aligned during the low deflection measurements–this eliminates error introduced by misalignment and side observation of the deflection. In Fig. 3a the perpendicularity error of the cantilever is evaluated to be > 1.5°. Alignment of the adhesive tape with the edge of the support chip is important to ensure correct anchoring of the cantilever, microscopy allows this to be ~ 100 µm accuracy. However, it is important that the flax fibre cantilevers be in plane with the surface of the support chips to ensure no pre-deflection of the flax fibre cantilever—this was ensured for all samples tested.

Figure 3b and c show top and side views of the cantilever. Digital optical microscopy was used to evaluate the fibre uniformity along the whole fibre length—this ensured the use of genuinely single fibres for the study. This provides a comprehensive view of the entire length of the fibre-based cantilever and includes zoomed-in images of its top, centre and bottom sections, each indicated by white rectangles. Figure 3c shows a side view of the cantilever's centre section. Microscopy (under different lighting conditions) also enabled the selection of cantilevers free of apparent ‘kink band’ defect features. The microscopy enables an accurate determination of the average fibre diameter and standard deviation; this is essential for a precise calculation of the flexural modulus using the model.

**Figure 4:**
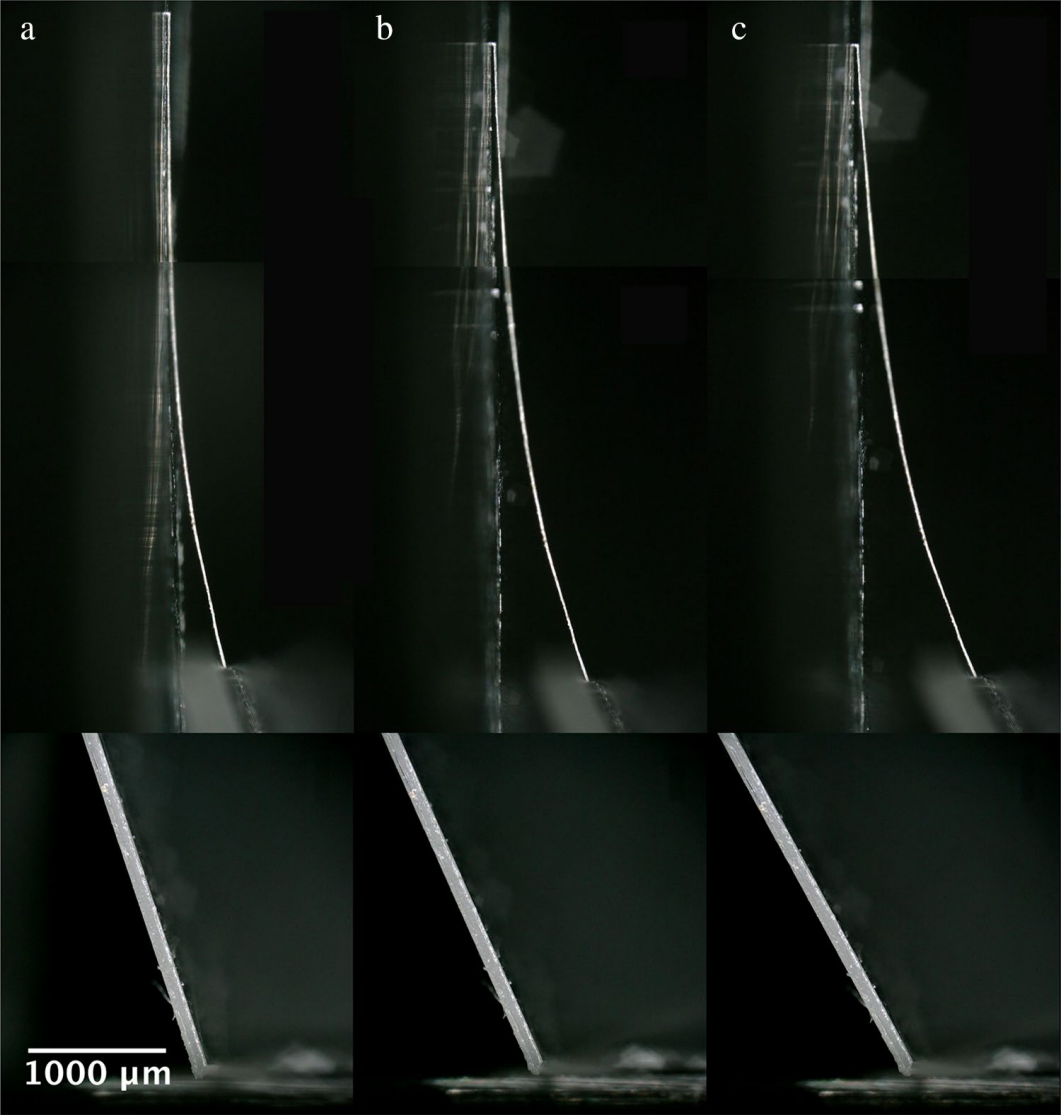
A single flax fibre based microcantilever in low deflection. The images (**a**–**c**) show how the deflection is increased by modifying the support chip angle. Upper images show the deflecting fibre, lower images show the support chip. The support chip leaning angle is (**a**) 71.7°, (**b**) 65.9°, and (**c**) 61.4°.

Next, the sample weights were evaluated (see “Methods” section) for two reasons. First, to meet the conditions of Eq. 11 (low-deflection regime) and Eq. 12 (tip-tangency condition). Second, to ensure that the fibre weight is negligible compared to the total sample weight. The cantilever weight is negligible compared to the support chip (see “Methods” section). Subsequently the samples were tested in low deflection using the method described above (see Fig. 1a and “Methods” section). Figure 4 shows an example of a single flax fibre cantilever being deflected using our micromechanical methods.

To obtain the images in Fig. 4 the sample is initially mounted to have a large value of theta. In this case the tip can be close to tangency with the vertical surface, see Fig. 4a. Next, the leaning angle is decreased to increase the force on the tip of the cantilever–as explained above. Increasing this force increases the tip deflection—this is recorded by tilting the digital optical microscope to 90 degrees relative to the sample. A large working distance coupled with high zoom (up to × 1000), and image stitching enables the whole of the deflected fibre to be recorded. The length of the cantilever is 4752.6 µm and the diameter is 18.5 µm. The length of the support chip is 7227 µm.

**Figure 5:**
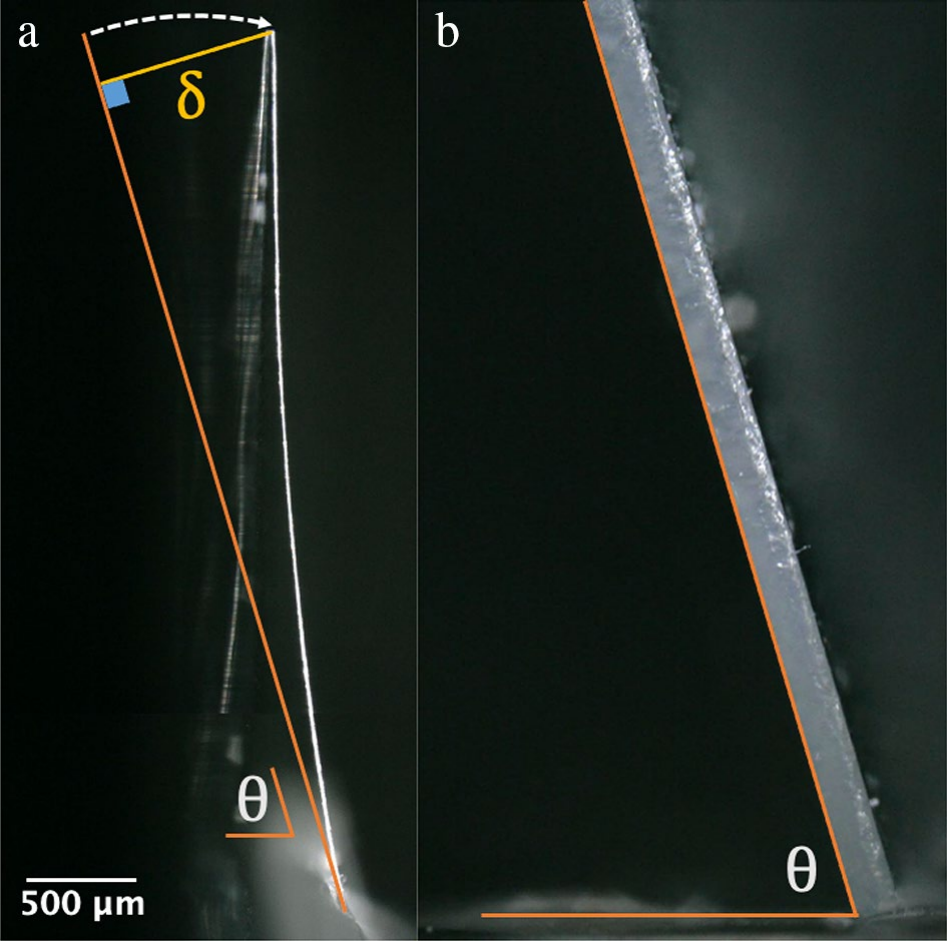
Analysis of the deflecting cantilevers. (**a**) Geometrical extraction of the cantilever deflection. (**b**) Photograph of the support chip angle.

In Fig. 4 the tilt angle is varied over a range of approximately 10°. This is enough to change the deflecting force and the observed deflection of the tip of the cantilever. As the tilt angle decreases, the deflection of the cantilever increases—see trend in Figs. 4a–c. Figure 5 shows how the deflection of the cantilevers was extracted from the experimental data.

**Figure 6:**
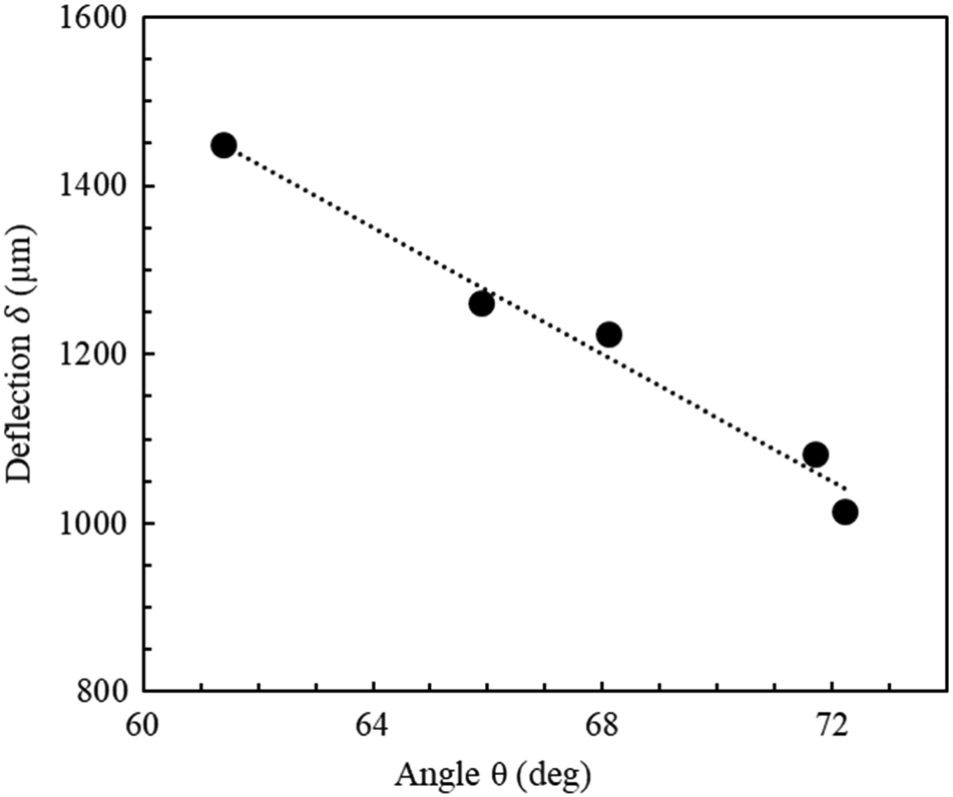
An example of a plot of the cantilever deflection as a function of the support chip angle. In this case the length of the cantilever length is 4752.6 µm, the average diameter is 18.5 µm, and the mass of the support chip is 4.034 mg. The length of the support chip is 7227 µm.

The orange line in Fig. 5a represents the initial state of the cantilever before any deflection occurs. The arc of the deflection of the cantilever tip is indicated by the white dotted arrow. The deflection distance is indicated by the yellow line, perpendicular to the initial non-deflected cantilever state represented by the orange line. Figure 5b provides a visual representation of how the leaning angle $\theta$ was measured. Figure 6 shows a graphical representation of an example of the data obtained from the experimentation.

**Figure 7:**
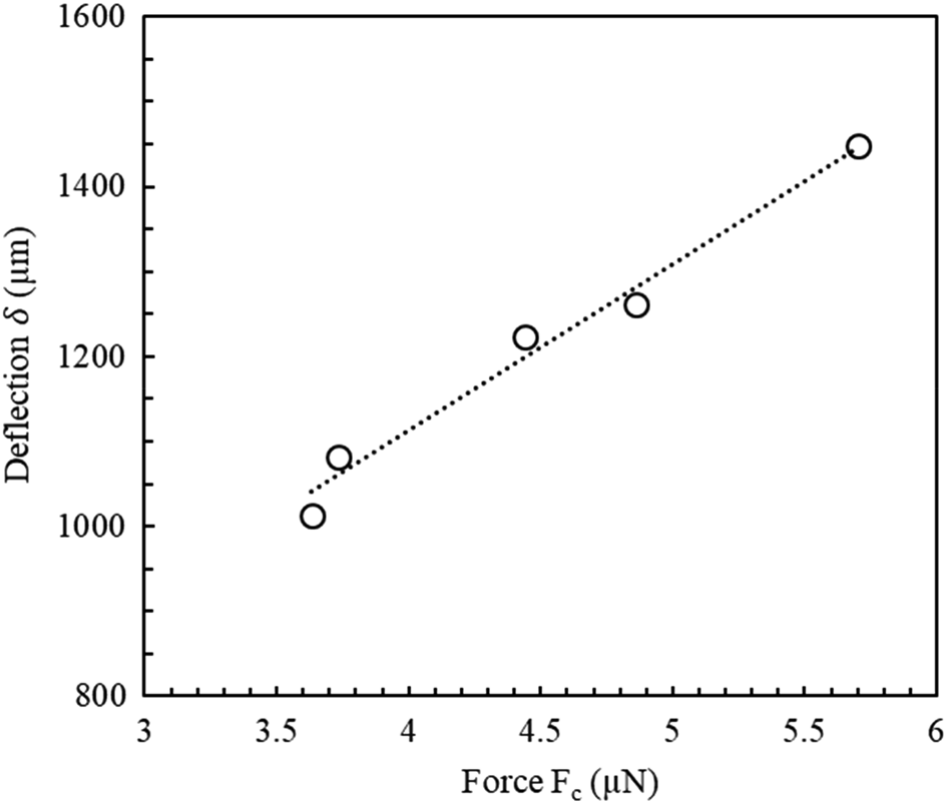
An example of a plot of the cantilever deflection as a function of the deflection force. In this case the length of the cantilever length is 4752.6 µm, the diameter is 18.5 µm, and the mass of the support chip is 4.034 mg. The length of the support chip is 7227 µm. The deflection force varies from 3.6 µN to 5.7 µN.

The figure shows the relationship between the angle of the support chip (varied by moving the precision linear stage) and the corresponding cantilever deflections—extracted from the microscopic images, as explained above. The support chip tilt angle was varied from 72.3° down to 61.4°. These angles resulted in a variation of the cantilever tip deflection of 1013.3 µm to 1447.3 µm. When plotted, the data can be fitted using a linear function (dashed line in Fig. 6). The slope is − 37.4 µm/degrees and the coefficient of determination is 0.98. Figure 7 shows the relationship between the cantilever deflection and the deflection force; the force is calculated using the tilt angle of the support using Eq. 6.

When plotted, the data in Fig. 7 can be fitted using a linear function (dashed line in Fig. 7). The coefficient of determination is 0.98. The slope of the plot is 260.46 m/N meaning that the inverse of the slope of this data, i.e. the stiffness of the cantilever, is evaluated to be 3.48 × 10^−3^ N/m.

However, before computing the flexural modulus the circularity and porosity of the fibres must be taken into consideration. There are two reasons for this: (1) the cross-sectional shape can differ from fibre to fibre and (2) lax fibres are known to have porosity (hollowness) known as a lumen^24,25^. Both these issues will affect the accuracy of the mechanical modelling if a cylindrical fibre approximation is considered—as do many authors^26,27^. Indeed**,** Hanninen et al.^28^ pointed out the potential impact of the lumen hollowness of the fibres on the mechanical properties.

**Figure 8:**
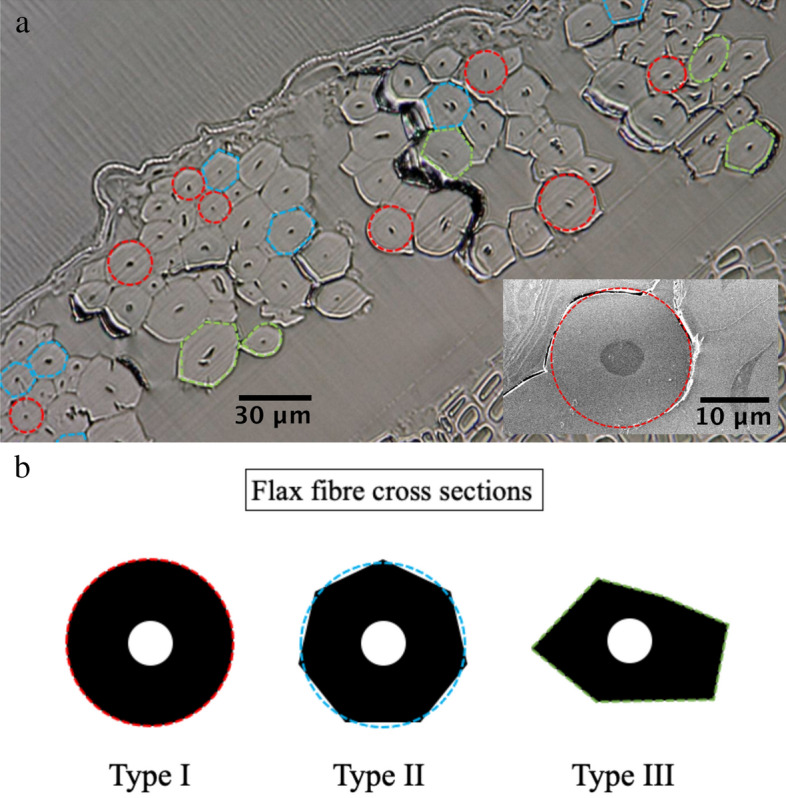
Images of stem cross section showing the fibres bundles. Images were obtained using (**a**) digital optical microscopy. The inset to (**a**) shows a scanning electron microscopy (SEM) image of the cross section of a single flax fibre. (**b**) Illustrations of the shapes of the observed fibre cross sections.

In order to assess these two issues, we have observed the cross section of flax stems used for the study. Cross sections of stem samples were prepared using resin embedding to provide mechanical rigidity for diamond sawing^29^. Figure 8 shows Images of the flax stem cross section and the fibre bundles.

First considering the circularity of the fibres. Figure 8a shows an example of images of a stem cross section taken using optical and electron microscopy (inset). Inspection of the experimental data revealed that fibres had three principal types of cross sections: (1) circular, (2) polygonal, and (3) irregular, see Fig. 8b. A proportion of the fibres shown in Fig. 8a have a near circular cross section (red shapes)—see also insert to Fig. 8a. There are several polygon-shaped cross sections with varying numbers of sides (blue shapes)—these can also be approximated to a near circular cross section for the modelling. However, there are also fibres with irregular cross sections (green shapes). These fibres were excluded from bending experiments using the top-view and side-view microscopy observations described above.

**Figure 9:**
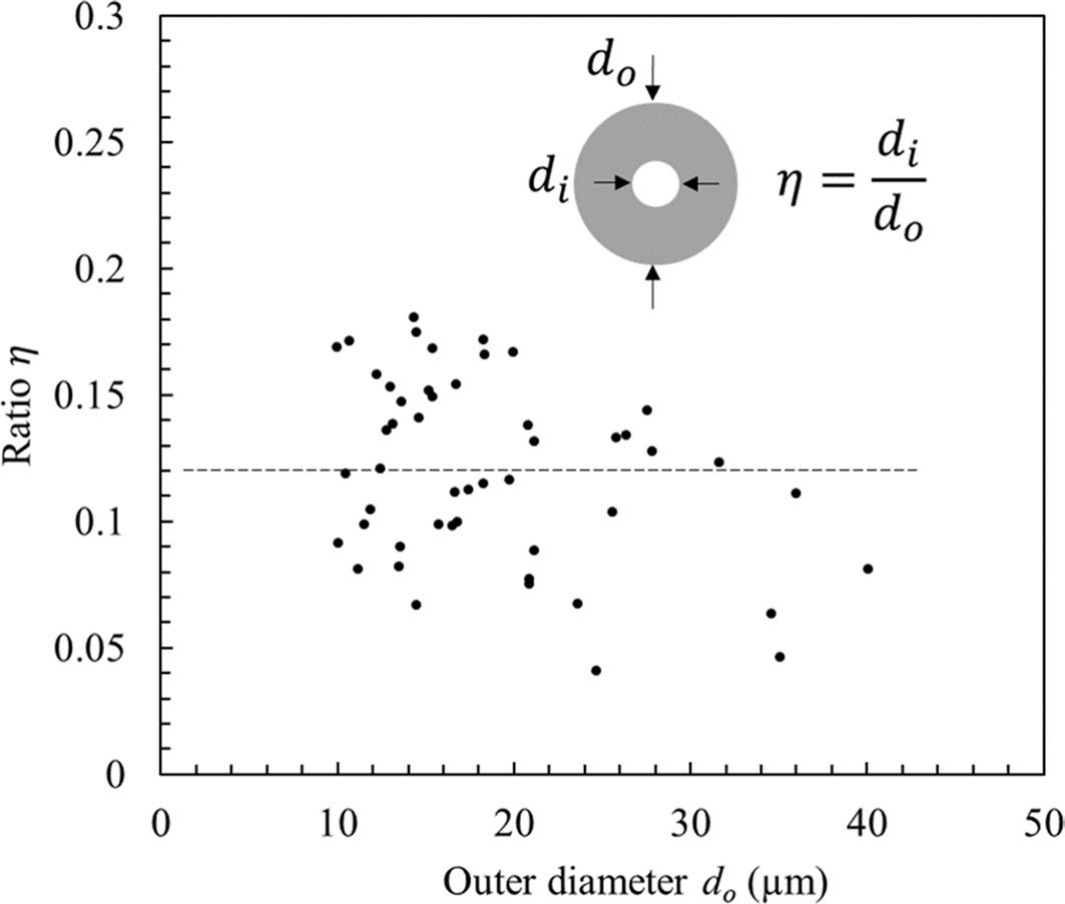
Plot of the ratio of the inner diameter to the outer diameter of flax fibres as a function of the outer diameter of the fibre. The inset shows a cross section of a flax fibre.

Second, considering the porosity of the fibres. A knowledge of the inner diameter is required for the modelling. However, As the measurement of the inside diameter is destructive we need a non-destructive method to evaluate the inner diameter. Cross sections data enabled the extraction of the inner/outer diameter ratio $\eta ={d}_{i}/{d}_{o}$ as a function of the outer diameter of the fibres to be plotted. Note that only near-circular cross section fibres were considered. Figure 9 shows a plot of the ratio *η* as a function of outer diameter.

Figure 9 was obtained using 50 fibre cross sections, and 12 diameter measurements per fibre cross section. The average value of $\eta$ was calculated to be 0.12 ± 0.04 for fibres with outer diameters ranging from 10 to 40 µm. Note that only near-circular cross section fibres were considered. Our findings agree with other published data^30^.

In the case of a porous fibre^24,25^ having an outer diameter ${d}_{o}$ and an inner diameter ${d}_{i}$, we have:

13 $${E}_{f}=\frac{32{L}_{c}^{3}mg{L}_{s}{\text{cos}}\theta }{3\delta \pi \left({d}_{o}^{4}-{d}_{i}^{4}\right)\left({L}_{s}+{L}_{c}\right)}$$

Taking the value of $\eta$ to be 0.12 from the experimental measurements, Eq. 13 reverts to Eq. 10, i.e. ${d}_{i}$ is negligible. Therefore, the flexural modulus ${E}_{f}$ can be written as:

14 $${E}_{f}=\frac{32{L}_{c}^{3}mg{L}_{s}{\text{cos}}\theta }{3\delta \pi {d}_{o}^{4}\left({L}_{s}+{L}_{c}\right)}$$

By using Eq. 14, we can now compute the flexural modulus of the fibres measured in the experiments. A summary of the low deflection results can be found in Table 1.

**Table 1:** Summary of results for the low deflection measurements.

Fibre test	Flexural modulus ${E}_{f}$ (GPa)	Deflection/length $\delta /{L}_{c}$
1	26.64	0.150
2	22.51	0.213
3	21.68	0.228
4	22.78	0.258
5	24.21	0.265
6	24.73	0.305
7	26.01	0.157
8	26.47	0.224
9	21.24	0.470
10	18.80	0.204
11	28.34	0.142
12	18.94	0.213
13	15.39	0.108

The average value of the flexural modulus of the fibres is 22.90 ± 3.70 GPa.

Based on 13 measurements, the value of the flexural modulus of these specific flax fibres was measured to be 22.90 ± 3.70 GPa. First, all the $\delta /{L}_{c}$ values are in the experimentally-valid range ($\le$ 0.3)^19–21^ except two (measurements 6 and 9). However, these two measurements give flexural modulus within the error. Second, concerning the geometric condition, two values (measurements 3 and 13) exceed this by more than 5%, but the computed flexural moduli are comparable with the other data. Third, this value is close to the result obtained from dynamic measurements (22.41 ± 6.06 GPa) of cantilever-based flax fibres collected from the same stem sample batch^23^.

The elastic modulus of natural flax fibre has been determined by several authors^8^, with most studies reporting the tensile modulus measured by tensile testing. The Young's modulus has been reported for different families of flax fibre in which our results are comparable with. For example, Charlet et al.^31,32^ reported a value of 58.1 ± 25.3 GPa for Hermes. Again, Charlet et al.^33^ reported 53 ± 25.6 GPa for Agatha variety. Alix et al.^34^ measured a value of 30 ± 11 GPa for ‘untreated’ flax fibre. Dittenber and GangaRao^35^ reported a modulus in the range 27.6–103 GPa. Pillin et al.^36^ measured the tensile modulus to be 48.0 ± 20.3 GPa. Soatthiyanon et al.^37^ reported a measured modulus of 19.4 ± 7.4 GPa. Arnould et al.^38^ report a modulus in the range 15–25 GPa using nanoindentation techniques. Aslan et al.^26^ measured the tensile modulus to be 31.4 ± 16.2 GPa.

Finally, this part of the study was very successful for the accurate measurement of the flexural modulus of single flax fibres with low dispersion results unlike previously reported values using tensile testing^8,26,37,39–43^. However, it is not able to produce enough deflection to be able to measure the flexural strength of the fibres. The flexural strength of the fibres is accurately determined in the next part of the paper by changing the measurement setup to be able to produce high deflection in the single fibre-based cantilevers.

## Measuring the flexural strength of a single fibre using a bending cantilever in high deflection

### Model

**Figure 10:**
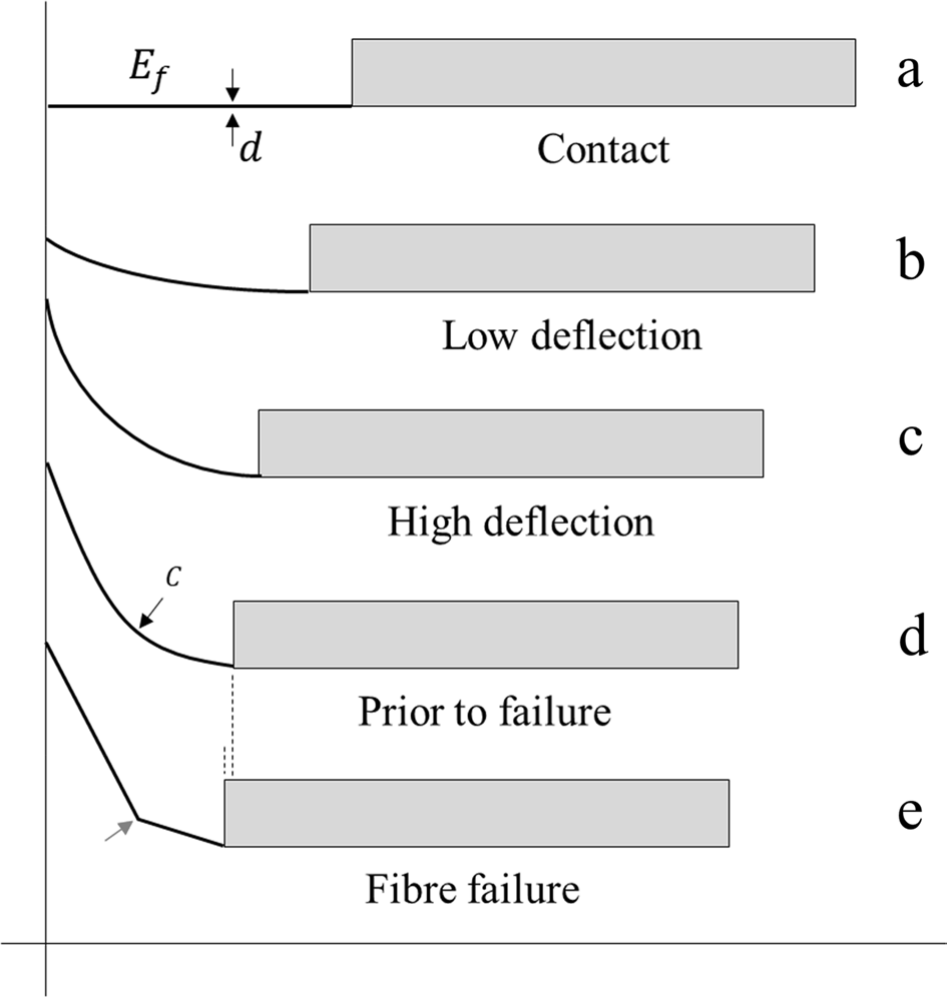
Schematic diagram of a single fibre-based cantilever sliding up a smooth surface. (**a**) Fibre contacts the surface, (**b**) Fibre in low deflection–tip slides up the surface, (**c**) Fibre in high deflection–increasing curvature in the fibre, (**d**) Fibre just prior to failure (black arrow), and (**e**) Local fibre failure (red arrow). The fibres have a diameter $d$, a flexural modulus ${E}_{f}$, and a local curvature $C$ .

This part of the study involves modelling of a deflecting single fibre-based cantilever to measure its flexural strength. Figure 10 shows a schematic diagram of the cantilever/support chip ensemble sliding up a smooth vertical surface.

First, the fibre is brought into contact with the rigid vertical surface—Fig. 10a. By moving the sample gradually using the precision linear stage, the cantilever deflects in low deflection by skating up the vertical smooth surface—Fig. 10b. By moving the linear stage further, high deflection of the fibre is achieved by further tip skating—Fig. 10c. The curvature of the fibre can be measured accurately using analysis of microscopy images (see “Methods” section)—taken prior to fibre failure—Fig. 10d. The red dashed line in Fig. 10 represents the point of failure. The top of the deflecting fibre is in compression whilst the bottom of the cantilever is in tension. Note that the support chip moves a short distance (dashed arrows) just prior to fibre failure, see Fig. 10d and e.

Let us now formulate a model to compute the flexural strength in terms of the local curvature prior to the point of failure. Based the reasoning of Sinclair^44^ and Fukuda et al.^45^, the surface strain $\varepsilon$ of a bending fibre in is given by the following relationship:

15 $$\varepsilon =\frac{dC}{2}$$

where d is the diameter of the fibre and C is the curvature (m^−1^) of the bending fibre.

The mechanical stress $\sigma$ is defined as^16^:

16 $$\sigma =\varepsilon E$$

where $E$ is the elastic modulus.

We can combine the above equations to give the flexural strength ${\sigma }_{f}$ as:

17 $${\sigma }_{f}=\frac{{d}_{o}{C}_{fail}{E}_{f}}{2}$$

where d_o_ is the outer diameter of the fibre—see above. ${C}_{fail}$ is the curvature at failure along the bending fibre at failure, and ${E}_{f}$ is the flexural modulus of the fibre.

## Experiments and results

**Figure 11:**
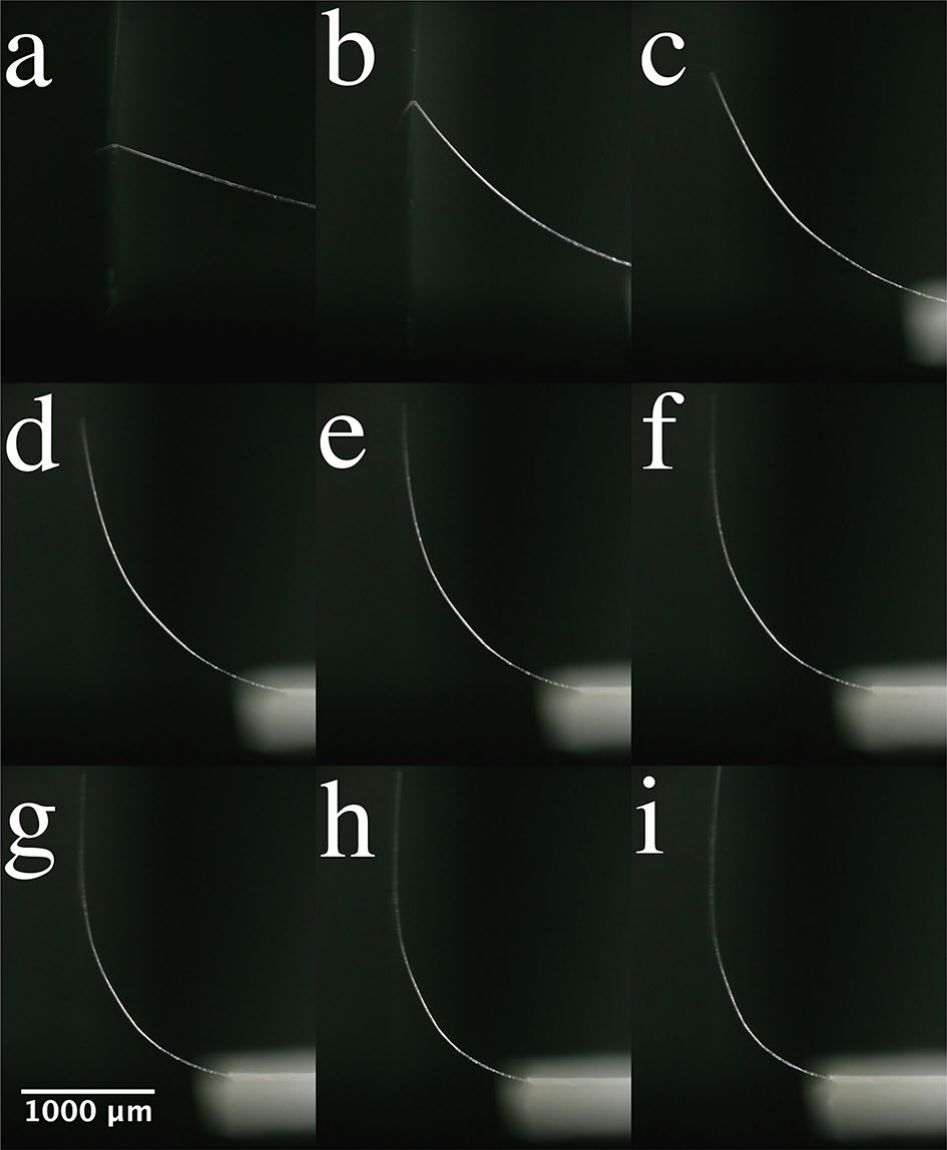
An example of a single flax fibre based microcantilever moving from low deflection to high deflection. The microscope images (**a**–**i**) show how curvature is increased by advancing the support chip towards the smooth vertical surface.

Fibre-base cantilever/support chips (as described above and methods) were deflected using the experimental set-up shown in Fig. 1b. The fibres were put into high deflection until failure and the deflection was observed using a digital optical microscope. Figure 11 shows an example of a flax fibre-based cantilever being put into a high deflection.

**Figure 12:**
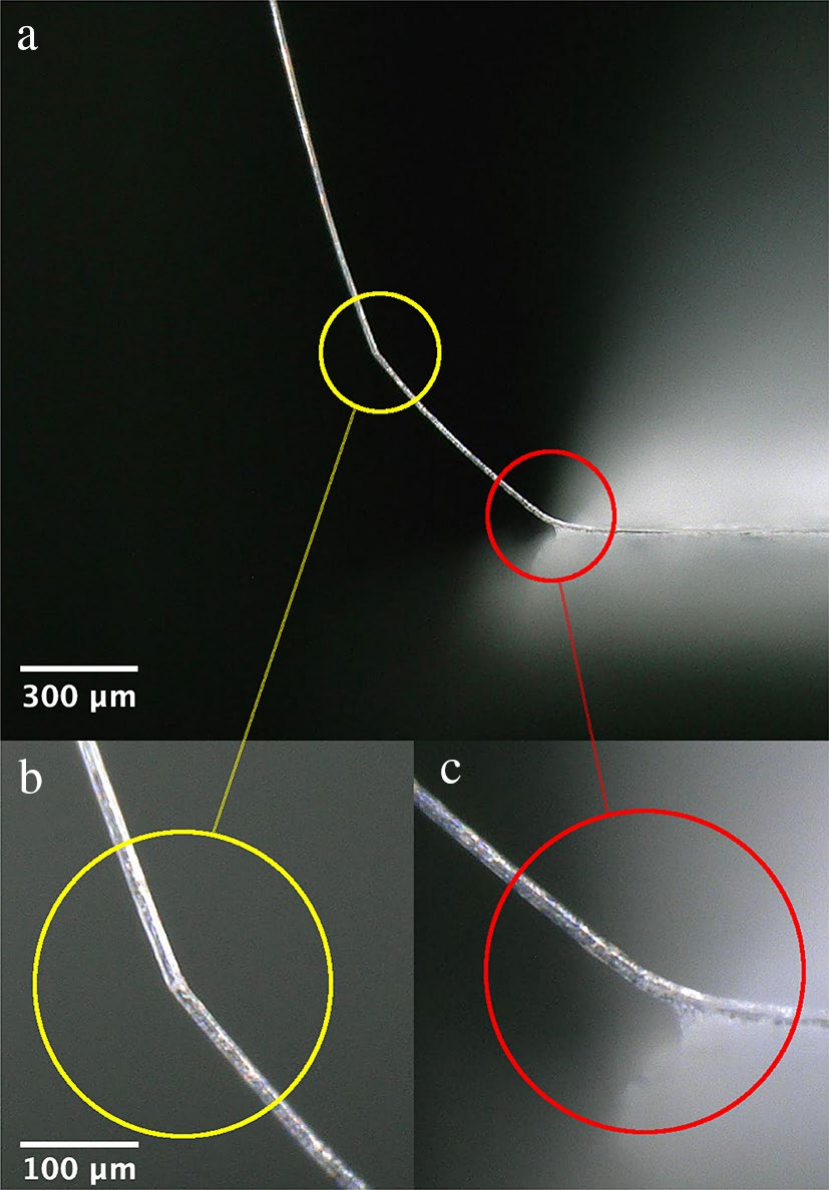
Examples of fibre failure. (**a**) Image of the whole fibre after failure. Zoom images of (**b**) Failure with kink band formation and (**c**) Failure without kink band formation.

One can observe the support chip (see at the bottom of the images) moving laterally towards the vertical surface, see Fig. 11a–i. One can observe the deflection increasing. This causes the curvature of the fibre to increase. Note that Fig. 11i is taken just prior to fibre failure. To ensure correct extraction of the curvature from the microscope images, two methods were used to vertically guide the tip of the fibre up the smooth surface (see “Methods” section). The curvature was extracted using software (see “Methods” section and Supplementary Information). Figure 12 shows fibre failure in high deflection.

Examples of the modes of failure observed in flax-fibre cantilever in high deflection are shown in Fig. 12. The observations suggest two distinct failure modes. One mode results in the formation of a visible kink band, see Fig. 12b (yellow circle). The other failure mode does not result in a visible kink band, see Fig. 12c (red circle).

Using the model, the flexural strength of both failure modes was determined through testing several samples. Table 2 summarised the results of these experiments.

**Table 2:** Summary of the experimental data gathered from the high deflection measurements of the flax fibre-based cantilevers.

Failure mode	Curvature ${C}_{fail}$ (µm)	Fibre diameter ${d}_{o}$ (µm)	S.D. ${d}_{o}$ (µm)	Flexural strength ${\sigma }_{f}$ (MPa)
Type A	0.0013	16.74	1.04	243.48
Type A	0.0014	15.34	3.94	239.05
Type A	0.0012	17.91	0.73	255.96
Type A	0.0012	17.55	0.56	242.80
Type A	0.0015	11.84	0.33	208.85
Type A	0.0009	18.45	0.77	191.46
Type A	0.0015	13.51	1.91	226.45
Type A	0.0011	18.46	2.06	223.28
Type A	0.0013	18.46	2.06	264.30
Type A	0.0012	12.67	0.76	171.84
Type A	0.0012	17.73	1.61	239.62
Type A	0.0016	14.81	1.39	265.89
Type A	0.0009	16.49	1.32	176.98
Type B	0.0022	17.80	0.62	442.75
Type B	0.0020	22.89	0.86	536.50
Type B	0.0019	22.89	0.86	503.22
Type B	0.0025	15.93	0.96	462.58
Type B	0.0013	30.63	2.33	451.80
Type B	0.0023	17.37	1.29	450.70
Type B	0.0047	9.02	0.66	483.38
Type B	0.0014	27.80	2.04	444.12
Type B	0.0023	18.45	0.77	492.60
Type B	0.0023	17.91	1.04	475.91
Type B	0.0020	21.41	1.17	490.31

Type A = fibre failure with kink band formation and Type B = fibre failure with no kink band formation.

**Figure 13:**
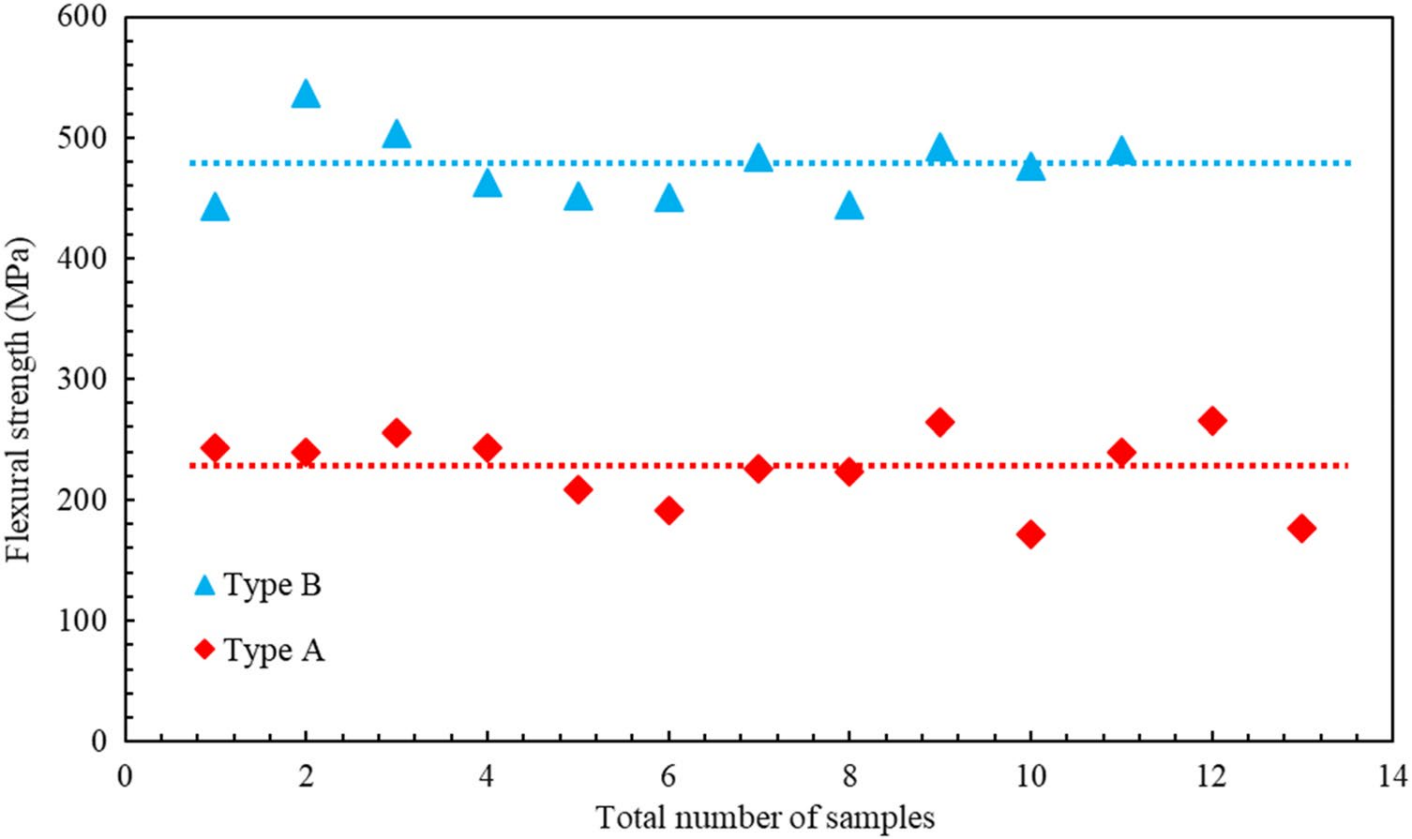
Graphical plot of flexural strength of the failure modes of the fibres. Type A (solid red diamonds) = fibre failure with kink band formation and Type B (solid blue triangles) = fibre failure with no kink band formation. The dashed lines show the average values.

The average outer diameters of the fibre and the corresponding standard deviations, shown in Table 2, were obtained from 15 measurements along the fibre length. Figure 13 provides a graphical representation that shows the flexural strength values for fibre failure.

These experiments yielded two distinct average flexural strength values corresponding to the two failure modes. Type A failure yielded an average value of the flexural strength of 226.92 ± 31.25 MPa and type B failure yielded an average value of the flexural strength of 475.81 ± 29.09 MPa. These average values are indicated by the dashed lines in Fig. 13. Let us now compare these results to others found in the literature.

First, in terms of the absolute values of the fibre strength. The strength of natural flax fibre has been determined by several authors^8^, with most studies reporting the tensile strength. For example, Soatthiyanon et al.^37^ measured tensile strength of 347 ± 136 MPa. Dittenber and GangaRao^35^ reported a tensile strength of 343–2000 GPa. Alix et al.^34^ found a tensile strength of 300 ± 100 MPa, and Aslan et al.^26^ measured a strength of 974 ± 419 MPa.

Second in terms of the failure modes. It has long been known that there are nodes^46,47^ (also known as ‘dislocations’^48,49^ and more recently ‘kink bands’^50^) along the length of flax fibres. These nodes are thought to be formed during plant growth or formed mechanically, e.g. through crop processing or manipulation/testing^51,52^. It has also been known for a long time that the nodes can be both visibly large and small^46,53^, and that these nodes affect the mechanical properties of the flax fibres^47^ and potentially composites made using flax fibres^41,54^.

More recently, and with the resurgence of the use of nature fibres, modern metrology has enabled more in-depth studies of such nodes and their formation^42,51^. Baley^55^ observed cracking in tension around kink bands using scanning electron microscopy, but tensile strength measurements yielded a high dispersion of results. A relationship of kink band and the mechanical properties of the fibres was observed. Bos et al.^56^ observed that kink bands become angular shaped when observed in microscopy—this is what we see here. They observed a difference in the compressive and tensile strengths of flax fibres^56^. This observation could explain why we see two values of flexural strength here and why we see two types of visible defect formation. They also stated that ‘sample length produces enormous scatter in tensile testing results’^56^. By using a loop test, the results suggested the compressive strength is less than the tension tensile strength. However, they observed that single, defect-free fibres displayed exceptional properties and that the employed processing methods used had a significant effect upon fibre properties. This agrees with our methods here where we are able to produce visibly defect-free fibres to form a cantilever which gives data with very low dispersion and distinct observations. Using atomic force microscopy, Melelli et al.^57^ reported no substantial differences in elastic modulus between the kink-band and defect-free regions, with average values ranging from 6.2 to 7.3 GPa. This measure shows that our use of the flexural modulus (obtained experimentally in low deflection) is valid to calculate flexural strengths of different failure modes. However, Melelli et al.^57^ reported no tensile strength measurements. Andersons et al.^58^ reported that kink bands are predominantly formed using compressive stresses. Thus, our observations of kink band formation are corroborated by this study^57^ and the observation of Bos et al.^56^ Using single flax fibres, Aslan et al.^26^ observed fracture surfaces of the flax fibres support a previously proposed failure mechanism of transverse failure followed by longitudinal splitting—i.e. different failure mode as we observe here. In addition to this, to achieve meaningful mechanical data, Aslan et al.^26^ noted that one requires near-circular cross section fibres—this is something our observation and measurement techniques enable.

Hanninen et al.^28^ used tensile testing to compare the mechanical strengths of flax fibres extracted from retted and non-retted samples. They found values of 487 ± 168 MPa and 322 ± 92 MPa for retted and non-retted respectively, which they attributed to the impact of biochemical activity of retting the external tissue. Their retted strength agrees well with our measurement of the flexural strength. Note also that our technique results in a lower dispersion of the data (six times less). Hanninen *at al*^28^ also measured the strength of flax fibres extracted using scutched and hackled techniques. Interestingly, they measure a value of 343 ± 144 MPa, lower than the retted fibres—our measurements here suggest that this observation is probably due to the presence of mechanically-induced kink bands in scutched and hackled fibre samples. Stems and fibres are known to have different failure modes^27,59^. Our observations concerning two types of fibre failure are in agreement with the ideas put forward by Ennos and Van Casteren^60^.

Finally, when comparing our results to those gathered using tensile methods, bending involves both tension and compression. A difference in the values of the compressive strength and the tensile strength has been observed in treated flax fibres^61^. Therefore, the flexural strength is not necessarily equal to the tensile strength.

## Conclusions

Original micromechanical measurements, inspired from microelectromechanical systems (MEMS) testing, can be used to yield accurate measurements of the flexural modulus and the strength of fibres without the need of a direct force measurement. These methods involve low and high deflection of single fibre-based microcantilevers. In addition, a micromechanical approach enables multiple measurements of the same sample—this is not possible using tensile methods. We were able to use single flax fibres to demonstrate the techniques. Like many fibres, flax fibres are rarely perfectly straight and often possess initial curvature. However, for the methods here to be valid, straight single fibres must be carefully selected. These techniques use small sample lengths which decreases the probability of the presence of defects in the fibre prior to testing. In addition, short samples ensure single fibres uniformity along the sample length. The techniques lead to low dispersion of results which have enabled an accurate determination of the average flexural modulus and strength values. The low dispersion of the experimental results has enabled us to identify and quantify two distinct failure modes in flax fibres. Finally, the generic techniques demonstrated here could be useful for the mechanical testing of a myriad of small fibres of restricted size and a valuable nature.

## Methods

### Flax sample gathering

Retted flax samples (Family: Linaceae, Genus: Linum, Species: L. usitatissimum, Variety: Felice) were collected for the study from a farmer’s field near Killem, France, run by a flax fibre manufacturer (Van Robaeys Frères. France). Sample gathering was conducted at midday after 6 h of sunshine (no rain) at an average temperature of 18.5 °C. To ensure a comprehensive representation, flax stalks were randomly selected from different parts of the field. The selected stalks were those that appeared healthy and undamaged. In order to protect the samples during transport to the laboratory, the flax stalks were enclosed in sealed plastic bags. These bags were then placed in a temperature-controlled container with ice to maintain their stability during transport.

### Single fibre extraction from flax stems

Upon arrival at the laboratory the stems (in their bags) were brought to room temperature for fibre extraction. A precise procedure was developed and followed to extract individual flax fibres. The flax samples were always taken from the middle of the whole flax plant stems. First, a slight break was carefully made at the top of the flax stem samples. This exposed the outer tissue of the stalk. This outer tissue was then carefully peeled away from the inner woody part of the stem without causing high bending of the skin/fibre mix. From the peeled outer tissue, a long and straight bundle of fibres was selected to be separate. This was carefully done using a combination of two pairs of tweezers to ensure that the fibres remain straight. Once a suitable fibre was identified within the bundle, the remainder of the bundle was peeled off while holding the individual fibre securely at one end using tweezers—again maintaining the fibre as straight as possible to avoid mechanical stresses. Note that the subsequent experimentation including the mechanical measurements were conducted in an ISO 7 class cleanroom (T = 20 °C ± 0.5 °C; RH = 45% ± 2%).

### Cantilever fabrication

To create the cantilever support, square polypropylene chips measuring (6 mm × 6 mm × 100 µm) were cut. These support chips had a mass of typically 4–7 mg. The individual flax fibre was picked up and transferred to the polypropylene support under the microscope using a small piece of adhesive tape measuring approximately 2 mm × 2 mm, assisted by tweezers. With the aid of a VHX-6000 digital optical microscope (Keyence, France) having a large working distance, we carefully applied the flax fibre/tape to the polypropylene support chip. The main aim here was twofold. First, the accurate anchoring alignment of the adhesive tape. Second, the perpendicular protrusion of the microcantilever (fibre) from polypropylene support. The adhesive tape was aligned with an accuracy of ± 100 µm. Finally, the mounted fibres were then examined using digital optical microscopy for perpendicularity, anchoring quality, and fibre homogeneity and uniformity.

### Sample weight

Following fabrication of the cantilever/support chip ensemble, the mass of each sample was accurately measured using precision scales (Mettler Toledo, France). These scales are accurate to 0.2 µg. The density of flax fibre is typically 1500 kg m^−3^^62–67^, if we consider the diameter of the largest flax fibres to be 40 µm and their length to be 5 mm, then its mass is 9 × 10^−9^ kg. The plastic support chips are typically 4 × 10^−6^ kg, giving a weight ratio of ~ 0.23%. The contribution to bending of the self-weight of the cantilever is therefore negligible—as required by the modelling.

### Experimental setup for low and high deflection bending: digital optical microscopy

A custom-built mechanical setup was used for the measurements. It consisted of a precision linear stage (displacement resolution = 10 µm) and a flat, vertically oriented, Teflon block covered by a grounded smooth aluminium foil^68^ to avoid electrostatic adhesion problems. In addition, grounded wristbands were employed during the measurements. The static coefficient of friction of flax fibre on smooth aluminium was evaluated to be between 0.23 and 0.27. For accurate measurement of the radius of curvature, two methods were employed to guide the fibre slipping up the vertical wall as the linear stage was used to increase its curvature. The first method used two vertical parallel tracks of thin tape (~ 30 µm thick). The second method involved manually orientating the fibre up the vertical surface using a tiny metal implement, when the fibre was in relatively low curvature. Once the fibre was orientated vertically, it maintained this state until failure.

### Image analysis: extraction of curvature

The radius of curvature was extracted from the microscope images using image analysis software (ImageJ).

### Ethics declaration

The authors acknowledge that they have complied with all relevant institutional, national, and international guidelines and legislation concerning the IUCN policy statement on research involving species at risk of extinction and the convention on the trade in endangered species of wild fauna and flora.

### Supplementary Information

Supplementary Information.

## Data Availability

The datasets used during the current study are available from the corresponding author on reasonable request.
