# Supplementary material for: Static micromechanical measurements of the flexural modulus and strength of micrometre-diameter single fibres using deflecting microcantilever techniques
Source: Sci Rep. 2024 Feb 5;14:2967. doi: 10.1038/s41598-024-53082-4 (PMC10844317; doi:10.1038/s41598-024-53082-4)
Supplement: Supplementary file 1 — Supplementary Information. [file 41598_2024_53082_MOESM1_ESM.pdf]

Supplementary Information for:

Static micromechanical measurements of the flexural modulus and strength of micrometre-diameter single fibres using deflecting microcantilever techniques

Ali Reda and Steve Arscott

University of Lille, CNRS, Centrale Lille, University Polytechnique Hauts-de-France, UMR 8520-IEMN, F-59000 Lille, France.

Tip tangency and the maximum δ/L ratio for a given angle θ

Supplementary Figure 1 shows the tip of the cantilever becoming tangential to the vertical surface for two different values of the support chip leaning angle θ.

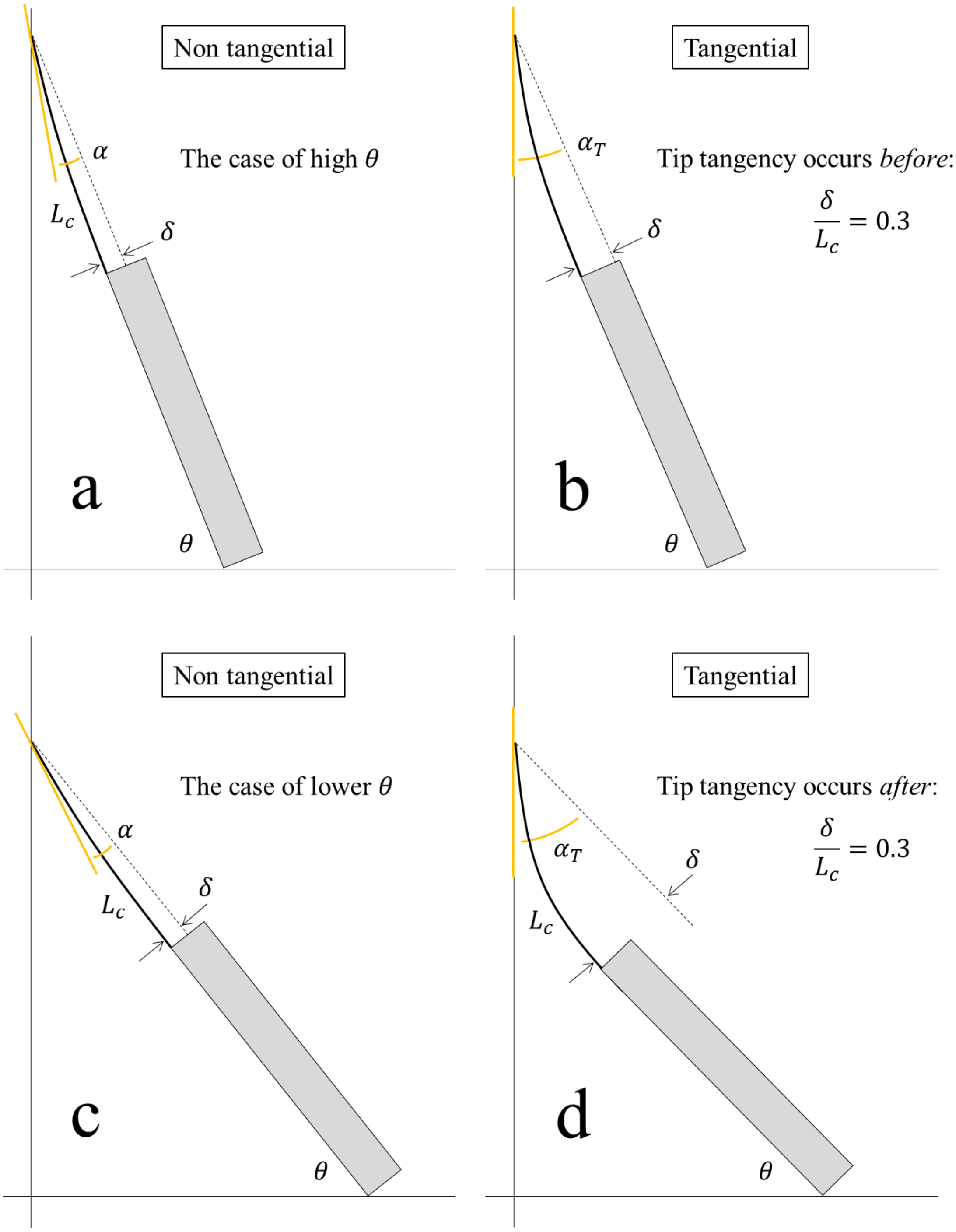

Supplementary Figure 1. Cantilever tip tangency. (a) and (b) the case of high values of θ. (c) and (d) the case of lower values of θ. In (a) and (c), the tip of the cantilever is not tangent to the vertical surface. In (b) and (d), the tip of the cantilever is tangent to the vertical surface.

Supplementary Figure 1a and Supplementary Figure 1b show how the tip becomes tangential to the vertical surface before δ/L_c = 0.3. Supplementary Figure 1c and Supplementary Figure 1d show how the tip becomes tangential to the vertical surface after δ/L_c = 0.3.

In the case of a cantilever having a constant cylindrical cross section, the bending angle α at the end of the cantilever is given by:[1,2]

$$\alpha = \frac{3}{2}\left(\frac{\delta}{L_c}\right) \qquad \text{(S1)}$$

Where δ is the tip deflection and L_c is the cantilever length—see Supplementary Figure 1.

The bending angle α_T for tip tangency with the vertical surface is given by:[3,4]

$$\alpha_T = \frac{\pi}{2} - \theta \qquad \text{(S2)}$$

where θ is the chip leaning angle, see supplementary Figure 1.

Therefore, for a given value of θ, the maximum value of the ratio δ/L_c is given by:

$$\left(\frac{\delta}{L_c}\right)_{max} = \frac{1}{3}(\pi - 2\theta) \qquad \text{(S3)}$$

As illustrated in Supplementary Figure 1, and depending on the different parameters, tip tangency can occur before or after δ/L_c = 0.3.

The case of zero wall friction

Supplementary Figure 2 shows the case of zero wall friction.

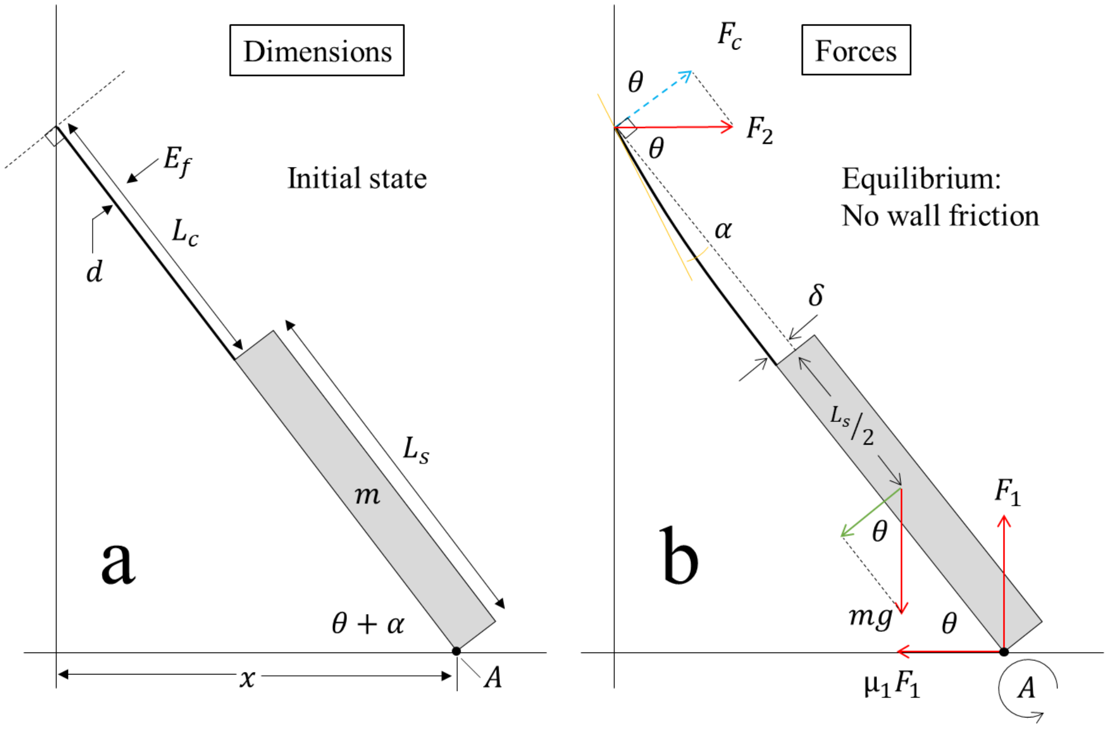

Supplementary Figure 2. The case of zero wall friction.

Considering Supplementary Figure 2, resolving forces vertically we have:

$$F_1 - mg = 0 \tag{S4}$$

Resolving forces horizontally we have:

$$F_2 - \mu_1 F_1 = 0 \tag{S5}$$

Taking moments about point A we have:

$$F_2 \sin(\theta)\,(L_s + L_c) - mg\cos\theta \frac{L_s}{2} = 0 \tag{S6}$$

$$F_2 = \frac{mgL_s}{2\tan\theta\,(L_s+L_c)} \tag{S7}$$

The bending force, perpendicular to the support chip, on the end of the cantilever can be written:

$$F_c = F_2 \sin\theta \tag{S8}$$

F_c is the force acting perpendicular to the cantilever direction—given by the dashed blue arrow in Figure 1b.

Therefore:

$$F_c = \frac{mgL_s \sin\theta}{2\tan\theta(L_s+L_c)} \tag{S9}$$

Which simplifies to:

$$F_c = \frac{mgL_s \cos\theta}{2(L_s+L_c)} \tag{S10}$$

For a cylindrical cantilever the relationship between the force of a concentrated load at the end of the cantilever F_c and the tip deflection δ is given by:[1,2]

$$F_c = \frac{3\delta\pi E_f d^4}{64L_c^3} \tag{S11}$$

Therefore, we can equate Equation 6 and Equation 7 to give:

$$\frac{3\delta\pi E_f d^4}{64L_c^3} = \frac{mgL_s \cos\theta}{2(L_s+L_c)} \tag{S12}$$

And the flexural modulus of the cylindrical fibre can be written as:

$$E_f = \frac{32L_c^3 mgL_s \cos\theta}{3\delta\pi d^4(L_s+L_c)} \tag{S13}$$

This is exactly the same result when considering wall friction, see Eq. (10) of the article.

The case of a rectangular cantilever

The authors suggest that the method could be applicable to other shape and size cantilevers.

For example. for the very common rectangular cantilever we have:

$$\frac{\delta Ewt^3}{4L_c^3} = \frac{mgL_s \cos\theta}{2(L_s+L_c)} \tag{S14}$$

And the bending modulus E of the cantilever is given by:

$$E = \frac{2L_c^3 mgL_s \cos\theta}{\delta wt^3(L_s+L_c)} \tag{S15}$$

The maximum stress on the surface of a bending rectangular cantilever (at the base of the cantilever) is given by:

$$\sigma_b = \frac{6F_c^b L_c}{wt^2} \tag{S16}$$

Where F_c^b is the equivalent force at the cantilever tip when breaking occurs. Substituting gives:

$$\sigma_b = \frac{3L_c mgL_s \cos\theta_b}{wt^2(L_s+L_c)} \quad \text{(S17)}$$

Where θ_b (the chip angle at cantilever failure) can be measured from the image.

Again, the measurements are independent of friction.

Fibre curvature extraction

Supplementary Figure 3 illustrates the process of the extraction of the radius of curvature. This is achieved by fitting a function to the bending fibre. The software then computes the curvature at the failure point along the bending fibre (see Methods).

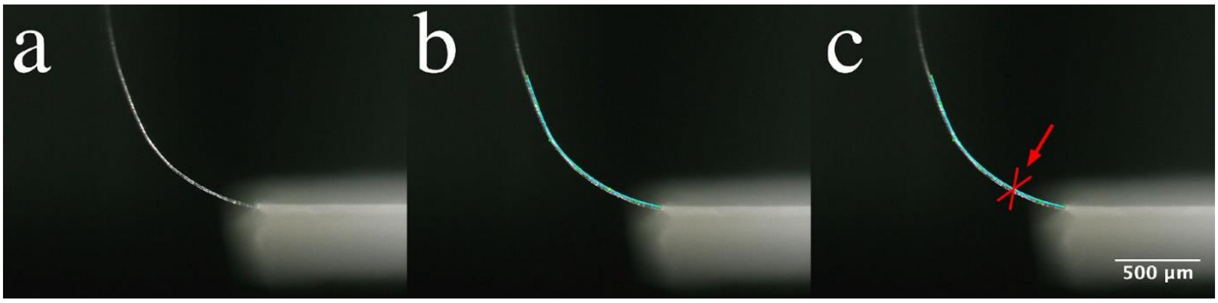

Supplementary Figure 3. Extraction of the curvature along the deflecting fibre-based cantilever. (a) Digital optical microscopy captures the fibre just prior to failure, (b) a curve is fitted to the bending fibre, and (c) the curvature is computed from the fitting function.

Examples of the two failure modes observed in the study

Supplementary Figure 4 and Supplementary Figure 5 show examples of the two failure modes observed in the study.

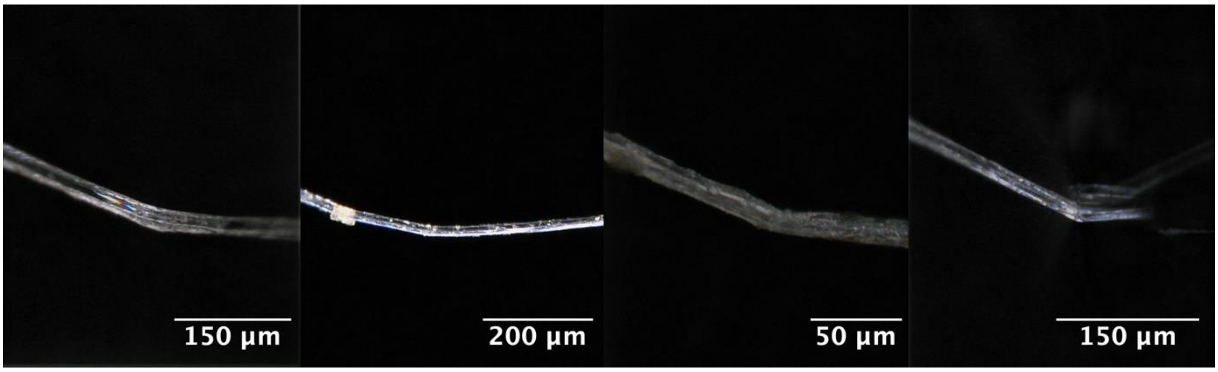

Supplementary Figure 4. Microscope images of examples of single flax fibre cantilever failure resulting in kink band formation.

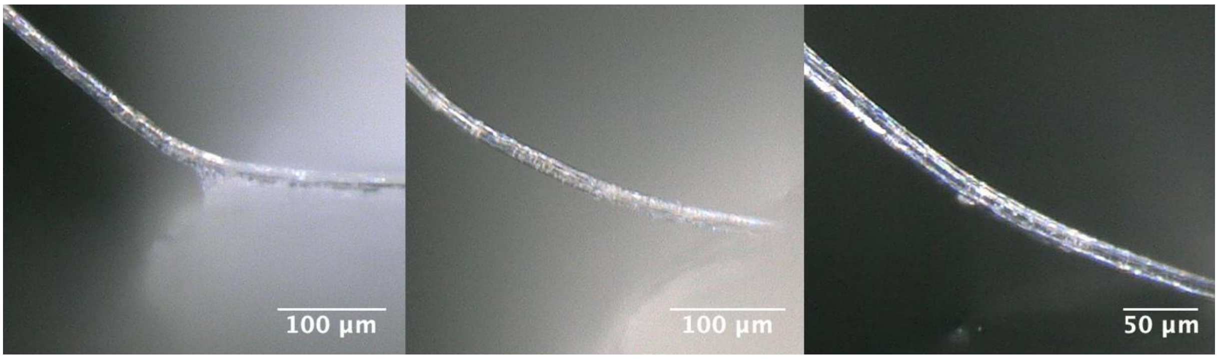

Supplementary Figure 5. Microscope images of examples of single flax fibre cantilever failure not resulting in kink band formation.

References

1. Timoshenko, S. *Theory of elasticity*. (McGraw-Hill Education, 2010).

2. Young, W. C. & Budynas, R. G. *Roark's formulas for stress and strain*. (McGraw-Hill, 2002).

3. Arscott, S. On overtravel and skate in cantilever-based probes for on-wafer measurements. *J. Micromech. Microeng.* **32**, 057001 (2022).

4. Arscott, S. Skate, overtravel, and contact force of tilted triangular cantilevers for microcantilever-based MEMS probe technologies. *Sci Rep* **12**, 19386 (2022).
